# Machine-Learning-Driven Stochastic Modeling Method for 3D Asphalt Mixture Reconstruction from 2D Images

**DOI:** 10.3390/ma18163787

**Published:** 2025-08-12

**Authors:** Jiayu Zhang, Liang Huang

**Affiliations:** School of Civil Engineering, Zhengzhou University, Zhengzhou 450001, China; zhangjiayu2022@stu.zzu.edu.cn

**Keywords:** asphalt mixture, 3D model, segment anything model, multiple-point statistics

## Abstract

Three-dimensional reconstruction programs are essential tools for understanding the behavior of asphalt mixtures. On the basis of accurate 3D models, it is convenient to identify the complex relationship between spatial structures and physical properties. In this work, we explore a low-cost and data-efficient way to create a collection of 3D asphalt mixture models. The core idea is to introduce a foundational segmentation program and stochastic modeling into the asphalt mixture reconstruction framework. First, our approach captures a 2D image to present spatial structures of the investigated sample. The integration of a smartphone camera and an image quilting method has been designed to understand fine-grained details and facilitate full coverage. Aiming at realizing high-quality segmentation, we propose the Segment Anything Model (SAM)-driven method to distinguish aggregate grains and asphalt binder. Second, Multiple-Point Statistics (MPS) is activated to build 3D models from 2D training images. To speed up the reconstruction step, we apply Nearest Neighbor Simulation (NNSIM) to improve pattern searching efficiency. Aiming at calculating 3D conditional probabilities, the probability aggregation framework is introduced into the asphalt mixture investigation. Third, our program focuses on the modeling evaluation procedure. Determination of a two-point correlation function, analysis of distance and a grain size distribution assessment are separately performed to check the reconstruction quality. The evaluation results indicate that our program not only preserves spatial patterns but also expresses uncertainty during the material production step.

## 1. Introduction

Asphalt mixtures are important materials in pavement construction and maintenance. In general, an asphalt mixture is composed of aggregate grains, asphalt binder and additives. The behavior of an asphalt mixture is a decisive factor for the durability, load-bearing capacity and environmental resistance of a road pavement [1,2]. A well-designed asphalt mixture plays an important role in minimizing pavement distress and developing long-lasting infrastructure [3].

In recent years, 3D asphalt mixture models have emerged as tools to analyze and optimize the performance of pavement materials [4,5,6]. Compared with 2D imaging methods and empirical testing, 3D models enable researchers to visualize and simulate the internal structure of asphalt mixtures. Based on numerous 3D high-quality models, a group of software systems have been developed to calculate the phase distribution, geometrical characteristics and physical properties of asphalt mixtures. It is convenient to explore the connection between grain distribution and mechanical behavior. Moreover, 3D asphalt models are supported by advanced imaging techniques and computational tools. The gap between laboratory research and real-world application is significantly bridged.

There are two main ways to facilitate 3D asphalt mixture models. The first method focuses on developing an imaging device to understand the intrinsic structures of asphalt mixtures. For example, X-ray Computed Tomography (CT) scanners have emerged as prevalent tools for producing high-resolution 3D models [7,8,9]. The core component of a CT imaging system is the direct control of X-ray beams through a material sample. A collection of cross-sectional slices is captured to explicitly express the aggregate geometry, binder coatings and void networks. As a non-destructive device, one advantage of a CT scanner is that it preserves a sample’s integrity. Therefore, analyses of one individual sample can be repeatedly performed under varying conditions. Furthermore, the benefits of 3D models and numerical simulation are combined to predict asphalt mixture behavior under real-world scenarios. On the basis of digital models, Finite Element Analysis (FEA) software Abaqus [10,11] and Discrete Element Method (DEM) software Partical Flow Code [12,13] are useful for simulating the mechanical response to traffic loads, thermal fluctuations and moisture invasion. Although CT scanners are widely used in the field of asphalt mixture sample analysis, their high cost is the main barrier to their extensive application and flexible deployment. On the one hand, existing CT methods require expensive equipment and laboratory settings. A high operational cost is necessary due to the energy consumption, device maintenance and specialized operator requirement. On the other hand, their limited scalability is not suitable for large-scale and repetitive experiments. For an individual sample, the scanning step generally takes several hours. During the reconstruction procedure, a significant period of time is required to complete the noise reduction, artifact correction and spatial alignment.

The second way to develop 3D asphalt mixture models is the image processing technique. Based on CT slices, image segmentation attempts to separate material phases [14]. Global thresholding, histogram analysis and watershed algorithms are designed to delineate voids and aggregates in asphalt mixture images. To enable the automated and data-driven method, machine learning programs are established to explore underlying patterns within complex structure datasets. For example, Support Vector Machine (SVM) [15] and Random Forest [16] are widely used to analyze large datasets of asphalt mixture properties. On the other hand, deep learning and computer vision have attracted considerable attention in the past ten years. Convolution Neural Networks (CNNs) have been developed to remove noisy instances and enhance image quality [17]. As a pixel-level method, the semantic segmentation technique involves partitioning the input image into several non-overlapping areas. The boundaries of voids and aggregates are drawn to understand the complex morphology. Moreover, the Generative Adversarial Network (GAN) provides a data-driven approach to the synthesis of 3D asphalt models [18]. The core idea is to create two CNN programs in an adversarial manner. In order to generate new images, the generator module pays attention to converting random noise into a desired output. By comparison, the discriminator continuously compares real data from the training dataset and fake data created by the generator network. The two networks are simultaneously trained in a zero-sum game. With sufficient training iterations, the generator is able to produce realistic images. However, the deep-learning-based GAN framework encounters two main fundamental challenges. First, the high demand for training images has a negative impact on GAN deployment. In reality, it is difficult to collect adequate 3D images. A limited number of training instances leads to model collapse and an overfitting problem. Second, the training procedure within the GAN framework takes considerable time to yield a converged generator network. In general, the training program initiates two GAN networks with random weights with the purpose of ensuring stability and balance. It is computationally intensive and time-demanding to enable GAN to understand task-specific data.

**Figure 1:**
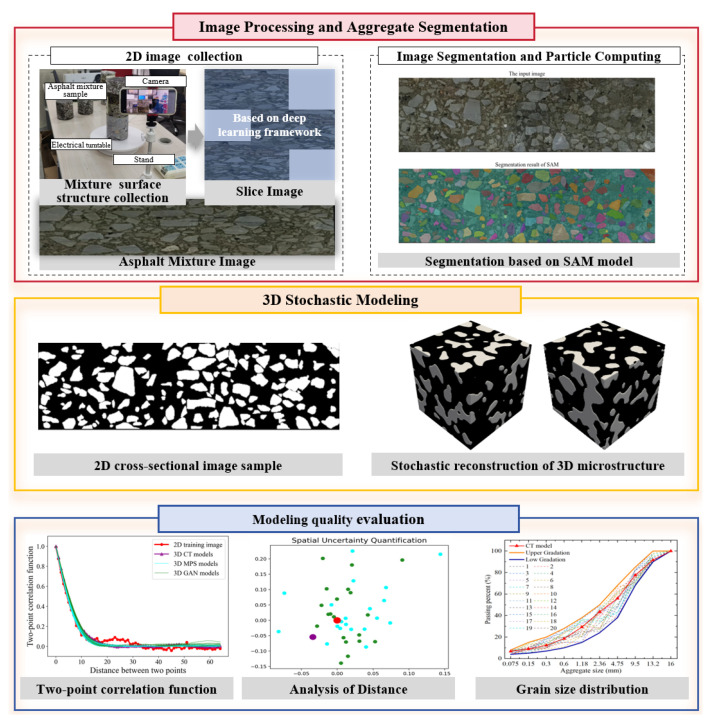
Basic workflow of our machine-learning-based stochastic modeling framework.

In this work, we focus on developing a low-cost way to generate diverse 3D models of asphalt mixtures. The strengths of the foundational segmentation method and 3D stochastic modeling are combined to create high-quality image datasets. As Figure 1 shows, there are three key steps. First, our program captures 2D images to express the spatial structure of asphalt mixture samples. A smartphone-mounted camera system is built to collect a series of multi-angle images. An image quilting program is conducted to stitch several overlapping regions into an individual image with fine-grained details and complete coverage. To highlight the target area, we launch the Segment Anything Model (SAM) to realize the semantic segmentation. The program automatically identifies aggregate grains and asphalt binder. Second, we perform Multiple-Point Statistics (MPS) to fulfill 3D stochastic modeling [19]. Based on 2D images, Nearest Neighbor Simulation (NNSIM) is carried out to store the training patterns and reproduce compatible structures [20]. A probability aggregation framework is introduced to recreate spatial patterns in the 3D simulation domain. Third, the modeling quality evaluation step pays attention to assessing 3D asphalt mixture realizations. Statistical functions are calculated to extract spatial correlation and dependency. According to the analysis of distance (ANODI) framework [21], the pattern reproduction ability and spatial uncertainty are individually examined. Grain size distribution is calculated to check the property of aggregation particles.

The main contribution of our program embodies the following three aspects.

• We explore a data-effective and low-cost way to generate high-quality 3D asphalt mixture models.
• With the objective to realize accurate semantic segmentation and grain identification, the powerful generalization ability of the Segment Anything Model is employed to tackle 2D asphalt mixture images.
• Based on 2D asphalt mixture images, Multiple-Point Statistics and Nearest Neighbor Simulation are employed to reproduce spatial patterns and generate diverse 3D models.

## 2. Image Processing and Aggregate Segmentation

The first step of our asphalt mixture modeling method is the image acquisition and processing of high-resolution 2D images. There are three basic modules. At first, a smartphone-based imaging system is designed to capture the spatial structure of the material surface. To tackle uneven illumination problems, the camera configuration is carefully specified to ensure image consistency. Then, we use an image quilting program to integrate several multi-angle images. With the purpose of conserving geometric integrity, a group of small images associated with limited viewpoints are combined into a unified large-size image. Finally, the pretrained SAM network is used to distinguish the aggregate grain and asphalt binder. As a foundational segmentation method, SAM has strong generalization ability to accurately identify target material components. The robust grain segmentation of SAM has a substantial influence on the structural characterization of heterogeneous asphalt materials.

### 2.1. Image Acquisition with Smartphone-Based Multi-Angle Imaging System

**Figure 2:**
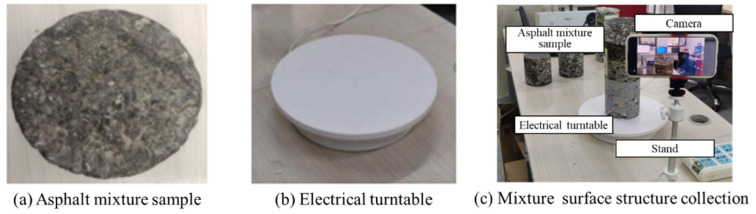
Basic modules of our smartphone-based imaging system.

Aiming at creating a low-cost method, we achieve the high-resolution 2D image acquisition of the asphalt mixture surface using a custom-designed smartphone-mounted camera system. This system takes advantage of the portability and image quality of modern smartphone cameras in a controlled experimental setup. The core of this setup involves the strategic placement of the asphalt mixture sample on a motorized electric turntable. Figure 2 shows the main components of our imaging system. At first, we place an asphalt mixture sample on an electric turntable. Next, a calibrated smartphone is positioned in front of the turntable. In this case, we specify the distance between the camera and asphalt sample as 30 cm to balance the spatial details and imaging coverage. A Xiaomi 14 smartphone, manufactured by Xiaomi Corporation, Beijing, China, and equipped with a Leica lens, manufactured by Leica Camera AG, Wetzlar, Germany, was applied to capture the fine-grain image. The camera configuration includes a 1/1.31-inch sensor and a resolution of size 4096 × 3072. The equivalent focal length for the main camera is 23 mm. Our program captures images under uniform and diffuse illumination provided by two daylight-balanced Light-Emitting Diode (LED) panels. To create multi-angle images at controlled angular increments, the turntable rotates the asphalt mixture sample through a full 360° trajectory. During the rotation movement, the calibrated smartphone systematically captures a series of overlapping 2D images of the asphalt mixture surface. For each sample, the turntable is rotated at a constant speed of 10 degrees per second. Eighteen high-resolution 2D images are systematically acquired at 20° intervals. Thus, a group of small images with overlapping areas provide complete coverage of the entire surface. This configuration builds a favorable balance between spatial completeness and computational efficiency. Adequate receptive field and structural details are explicitly expressed by 2D images.

To mitigate environmental disruption, we standardize the imaging circumstance using diffuse ambient lighting. The phenomena of specular reflections and glare are minimized to remove artifacts. The smartphone camera is calibrated to ensure coherent focal length, aperture and exposure settings across all captures. To optimize the tradeoff between spatial resolution and receptive field, we utilize a fixed distance of 30 cm between the camera and sample surface. An automated scripting module controls the rotation movement of our turntable. During the imaging process, manual intervention is eliminated to enhance reproducibility.

The camera images are stored in a lossless format. The fine-grained texture details of aggregates and binders are preserved. To detect the outliers of brightness and contrast, we conduct histogram analysis to realize the preliminary quality assessment. This systematic imaging system ensures that we capture the spatial structure of the asphalt mixture sample in 2D projections.

It should be noted that the lighting conditions and camera configuration play an essential role in our imaging system. On the one hand, our system is designed to mitigate the impact of lighting variations and shadows through a controlled illumination setup. To control image variability, it is necessary to have an appropriate lighting setting. An unsuitable light source has a negative impact on the image quality. On the other hand, the high resolution of a smartphone camera is a contributing factor. To ensure computational efficiency, it is helpful to find a balance between fine-grain details and image coverage. For aggregate grains of small size, it is difficult to find the boundary on the basis of a low-resolution image.

### 2.2. Large-Size Surface Image Synthesis via Deep-Learning-Based Image Quilting

Following the image acquisition step, the next technical problem is to create a comprehensive view of the mixture example. An individual image is produced to explain not only the entire coverage but also the structural details. In this case, we attempt to synthesize 18 localized images into a unified composite with an image quilting program. Originating from the texture synthesis community, the image quilting technique concentrates on stitching several overlapping image patches. The traditional quilting method is reliant on finding the patch boundaries. A graph-cut approach is implemented to minimize visible seams and geometric distortion. However, asphalt mixture samples bring a substantial challenge. The image quilting program inspired by the digital image processing technique struggles with the complex aggregate geometry and non-repetitive textures.

**Figure 3:**
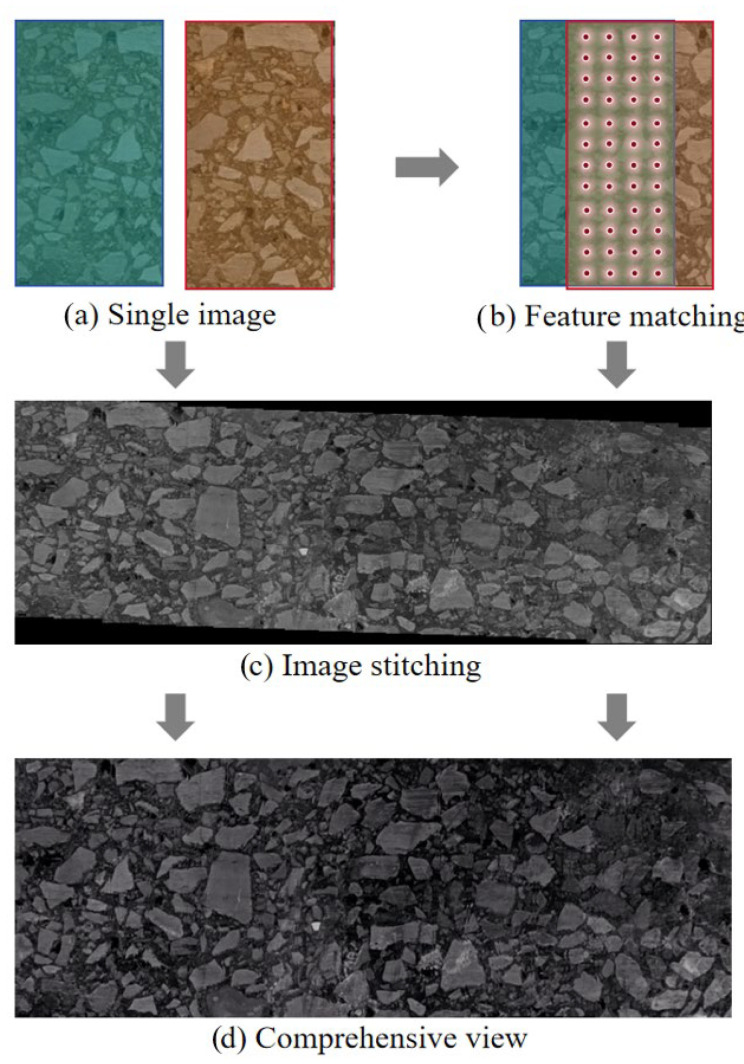
The workflow to image quilting program.

The image quilting method used in this study is based on the approach implemented in ImageJ version 1.54f. As shown in Figure 3, this image quilting method utilizes the Fourier shift theorem to compute all possible translations between 3D image pairs and determines the optimal overlap through cross-correlation [22]. Thus, the global configuration is optimized for the entire set of 3D images. Unlike traditional step-by-step registration techniques, this method minimizes error propagation with the intention of improving stitching accuracy. To address intensity differences between tiles, smooth nonlinear intensity transitions are applied to the overlapping regions, resulting in a more seamless stitching process.

### 2.3. Automated Grain Segmentation with Segment Anything Model

On the basis of a reliable 2D surface image, we focus on identifying material components as well as distinguishing the aggregate grain and asphalt binder. The segmentation step plays a key role in 3D modeling program due to the importance of characterizing the spatial structures of asphalt mixture samples. A clear identification of material components is beneficial for explaining the grain distribution. One primary challenge of asphalt mixture segmentation lies in the texture diversity between aggregates and asphalt binder. On one hand, aggregate particles experience significant changes in color, roughness and reflectivity. On the other hand, the asphalt binder is commonly presented as a dark and homogeneous phase.

To motivate the pixel-level separation of aggregate particles, we introduce the Segment Anything Model (SAM) into the asphalt mixture investigation. As a versatile deep learning technique, SAM was designed by Meta AI in 2023. The core idea of SAM is to establish a high-quality and prompt-driven segmentation framework. With the objective of overcoming the overfitting problem, SAM attempts to remove the task-specific network training step and build a general-purpose foundation program. The advantages of SAM embody the following three key points. First, the promptable segmentation is designed to facilitate practical applications. Different from previous deep-learning-based segmentation approaches, SAM is capable of guiding the calculation result by user prompts. For example, the positive point can be used to indicate the desired area and visual content. By comparison, irrelated regions are removed by negative points. Moreover, the bounding box becomes a feasible tool to encompass the image region of interest. The interactive and promptable characteristics enable SAM to avoid the network fine-tuning step, speed up the analysis process and improve the segmentation accuracy. Second, Vision Transformer (ViT) becomes the backbone module within the SAM framework. Compared with a Convolutional Neural Network (CNN), ViT utilizes the self-attention mechanism to capture the global trend and local characteristics within an input image. To observe the input image from varying perspectives, multi-head attention is employed by ViT. A wide range of real-world applications confirm that ViT has a strong ability to understand complicated visual content and motivate fine-grained downstream tasks. To ensure accurate delineation of the aggregate boundary, the ViT architecture encourages SAM to explore the intricate relationship between aggregate particles and asphalt binder. Third, the generalization capability of SAM is enhanced by the large-scale training strategy. As one of the biggest datasets in the field of semantic segmentation, the SA-1B dataset includes 11 million images and 1.1 billion high-quality masks. The image annotations are collected from a variety of domains such as natural scenes, industrial materials and microscopic images. The diverse training images inspire SAM to be robust to texture variability, low inter-class contrast and visual ambiguity.

**Figure 4:**
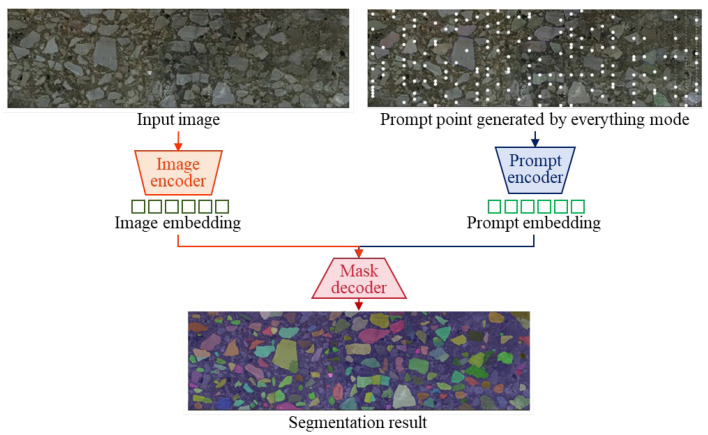
Basic components of SAM segmentation program.

As Figure 4 shows, the SAM program comprises three main modules. (1) Based on the ViT architecture, the image encoder network pays attention to analyzing the input image. A high-dimensional feature vector is produced to represent the visual content. In the feature space, the images with similar spatial structures share the close feature vectors. (2) The prompt encoder module converts the user-defined prompts into a high-dimension embedding. The input prompt plays a key role in guiding the SAM segmentation result. (3) The mask decoder network is dedicated to generating the final segmentation mask. Based on compatible image features and prompt information, the aggregates and asphalt binder are separated at the pixel level. The ability of the decoder has a substantial effect on the flexibility and robustness of the SAM program.

**Figure 5:**
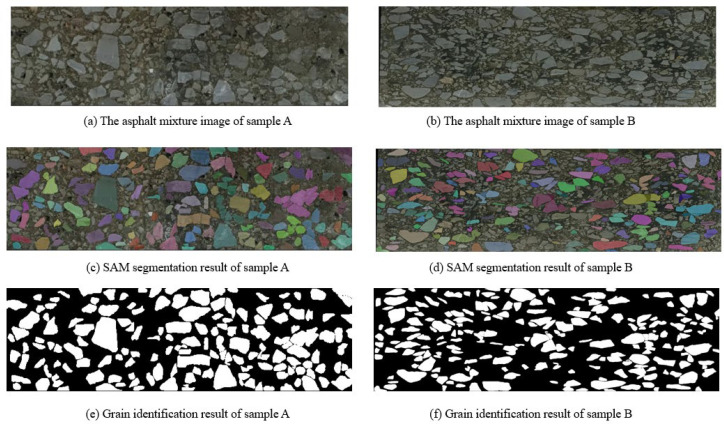
Segmentation results of SAM program.

In this case, we activate the everything mode of SAM to comprehensively handle the asphalt mixture image. As shown in Figure 5, two asphalt samples are utilized to validate our SAM program. Sample A corresponds to an asphalt concrete with a 13 mm nominal maximum aggregate size (AC-13) mixture, while sample B represents an asphalt concrete with a 10 mm nominal maximum aggregate size (AC-10) mixture. These two types exhibit distinct aggregate gradations and morphological characteristics, with AC-13 generally containing coarser aggregates compared to the finer gradation of AC-10. It is worth noting that one important benefit of the everything mode is to automatically generate a complete set of segmentation masks. The entire input image is partitioned into several non-overlapping areas. At first, the image encoder network is devoted to converting the input asphalt mixture image into an informative feature vector. Then, the dense mask prediction is realized by the prompt encoder. Without prior guidance, the program identifies all potential object boundaries. Next, a group of segmentation masks are generated on the basis of the mask decoder module. In order to remove the redundant and fragmented masks, non-maximum suppression and mask filtering steps are conducted to refine the segmentation mask.

Following the execution of SAM segmentation, we independently analyze each mask to determine the category of each material composition. In our grain identification program, the core concept is to calculate the average of and variance in pixel intensity within each mask. These low-level visual features serve as an indicator of texture of color uniformity. Based on the color information, the computer classifies a segmentation mask into an aggregate and asphalt binder category. In Figure 5, we highlight the occurrence of aggregate particles. According to human inspection, the SAM segmentation result provides a valuable insight into the microstructure characteristics of asphalt materials. In addition, it is worth noting that the SAM segmentation result is fed into the subsequent reconstruction module. The segmentation map becomes the prior knowledge to express the desired structures. Thus, segmentation errors might lead to inaccuracy in the 3D models. In order to address the error propagation issue, we conduct manual correction on the SAM segmentation result. Small-size aggregates are carefully delineated to express fine-grained structures.

**Figure 6:**
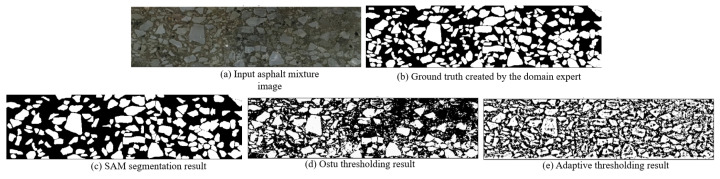
The segmentation result comparison of asphalt sample A.

**Figure 7:**
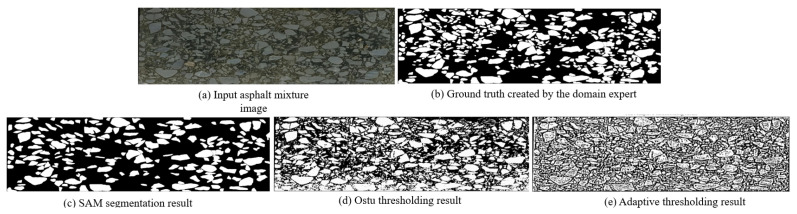
The segmentation result comparison of asphalt sample B.

One key innovation of our SAM-driven program is that there is no network training and image labeling step. Based on a pretrained weight, SAM is activated to tackle the asphalt mixture image. Our program identifies the aggregate grain according to the pixel intensity of each mask. In Figure 6 and Figure 7, we display the segmentation result conducted by Ostu thresholding and adaptive thresholding programs. It is clear that the thresholding methods do not yield favorable results. The main reason is that the digital image processing technique is heavily dependent on the pixel intensity. It is challenging to find a suitable boundary to separate two material components. A complicated dependency exists between aggregate grains and asphalt binder. In our work, the computer activates the powerful SAM network to analyze the image and create high-quality masks. The introduction of a foundational segmentation model has a helpful effect on the grain identification task.

Moreover, quantitative evaluation is carried out to check the segmentation accuracy. There are two primary steps. At first, the domain expert focuses on analyzing the asphalt mixture images and delineate the aggregates. Based on careful manual inspection, a segmentation map is created as the ground truth. Next, four evaluation metrics are employed to compare the human annotation and segmentation results, as shown in Figure 6. The computer individually calculates the Intersection of Union (IoU), dice coefficient, pixel accuracy and Hausdorff distance. According to the calculation results in Table 1, it is obvious that the proposed SAM program has a strong ability to identify aggregate grains from the input image.

The segmentation performance of the SAM model was comprehensively compared with traditional methods, including Otsu and adaptive thresholding, across multiple evaluation metrics. For both sample A and sample B, SAM consistently outperformed the other methods in terms of Intersection of Union (IoU), dice coefficient, pixel accuracy and Hausdorff distance. In sample A, SAM achieved the highest accuracy (94.56%) and dice coefficient (0.9379), along with a significantly lower Hausdorff distance (7.07), indicating a high degree of spatial agreement with the ground truth. Although the IoU (0.8830) was slightly lower than the dice score, this can be attributed to the blurred boundaries of aggregates caused by smartphone image acquisition and stitching artifacts, which introduced subtle discrepancies at the object edges. In sample B, despite an overall drop in performance due to more complex boundary conditions or lighting variations, SAM still showed superior results, particularly in terms of the dice coefficient (0.9204) and pixel accuracy (94.42%), with a notably lower Hausdorff distance (20.62) compared to the threshold-based methods. These results demonstrate that SAM provides robust and accurate segmentation even in the presence of imperfect image quality, while traditional methods exhibit sensitivity to noise and fail to capture fine boundary details effectively.

## 3. Three-Dimensional Stochastic Modeling from Two-Dimensional Training Image

In Section 2, we concentrated on image acquisition and aggregate segmentation tasks. A segmented 2D image is created to reflect the grain distribution of an asphalt mixture sample. In this section, the main research problem is to build 3D models on the basis of 2D training images. Therefore, we introduce the stochastic modeling framework into the asphalt mixture research. As a promising technique in the context of geoscience and porous material, we use a Multiple-Point Statistics (MPS) program to extract and reproduce spatial patterns in a 3D grid. Nearest Neighbor Simulation (NNSIM) focuses on accelerating the pattern searching step and saving running time. A probability aggregation framework is employed to synthesize 3D structures on the basis of multiple 2D patterns.

### 3.1. Principles of Stochastic Modeling and Multiple-Point Statistics

One key innovation of this work is to introduce stochastic modeling into the asphalt mixture investigation. In the context of porous material, randomness and uncertainty are at the heart of our understanding of material design and production. Therefore, the stochastic modeling technique is dedicated to generating multiple realizations of a spatial phenomenon. The biggest benefit is that the stochastic models describe a wide range of scenarios using the probability distribution and confidence interval. The uncertainty quantification [23] becomes a core concept because the material heterogeneity and data scarcity highlight the demand of probabilistic assessment. There are two main concepts within the stochastic modeling framework. On the one hand, spatial pattern reproduction ability emphasizes the similarity between training images and generated realizations. On the other hand, spatial uncertainty and diversity ensure that there is an intensive difference between two 3D models.

**Figure 8:**
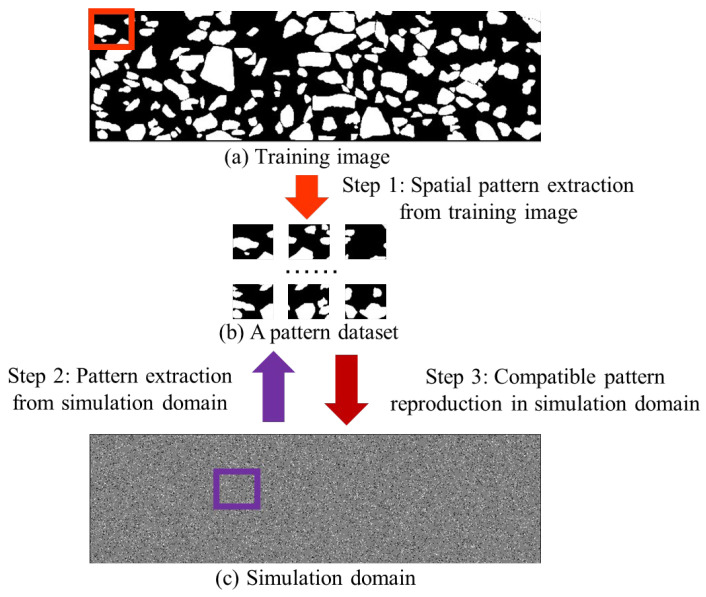
The core concept of MPS within the asphalt mixture investigation.

As an emerging method in the field of stochastic modeling, Multiple-Point Statistics (MPS) has gained considerable attention from scientific researchers and the engineering community. The main advantages of MPS are that a training image becomes the prior knowledge to explicitly express the spatial phenomenon of interest. Based on a training image, MPS attempts to capture geometrically complex patterns by simultaneously considering multiple points. As Figure 8 exhibits, there are three main steps within the MPS framework. First, the program employs a template to extract spatial patterns from training images. A square or circle is commonly used in the general-purpose scenario. To extract complicated structures, researchers explore templates with irregular shapes and flexible conditioning points. A collection of training patterns is extracted by checking every feasible point in a training image. The program builds a dataset to store all training patterns in computer memory. Second, the modeling procedure concentrates on generating new realizations. Based on the template, the computer visits an unknown point in the simulation domain. A conditioning pattern is created to explain spatial structures in the neighborhood. As the core component, a searching program is launched to find matching instances to the conditioning pattern. The Hamming distance is widely used to deal with the segmented image and categorical variable. In comparison, the continuous variable is tackled by the Euclidean distance. Third, the center point of compatible training patterns becomes the prediction result of the unknown point. In this case, a random sampling program is used to select one candidate point from multiple matching patterns. The point simulation program mentioned above is repeatedly performed until every point in the simulation grid is informed.

Compared with traditional two-point statistics, the main advantages of MPS embodies the following two aspects. On the one hand, the training image is an explicit tool to express the spatial structure under research. The accessibility of training images remarkably facilitates the practical applications of MPS. In recent years, the applications of MPS have grown into water resource research, petroleum engineering, mineral deposition and porous material. By comparison, it is not easy to understand the variogram in two-point statistics. On the other hand, MPS predicts the value of unknown points according to a group of neighboring points. The introduction of spatial patterns has a positive effect on reconstructing realistic structures. The pattern reproduction ability is important to simulate the complex structure and connectivity.

Moreover, deep learning and computer vision techniques have become a prevailing choice in the context of stochastic simulation. For example, Convolutional Neural Network (CNNs) are explored to extract high-dimensional features from input images. Generative Adversarial Network (GANs) attempt to produce high-quality realizations in accordance with training images. Compared with deep learning methods, MPS has three main benefits. (1) MPS has a low demand on training images. In many cases, an individual training image is used to inspire MPS programs. The main reason is that MPS focuses on reproducing spatial patterns from a training image to the simulation domain. It is robust in dealing with limited training data. In comparison, GANs rely on large and diverse datasets to learn the underlying data distribution. In reality, it is challenging to collect sufficient training images. (2) A lot of research effort has been made to speed up MPS modeling. For instance, data structures including search trees and lists have been explored to save running time. The direct sampling (DS) [24] method removes the training dataset module and performs the pattern retrieval procedure in the training image. By comparison, the deep neural network is trained from scratch in the GAN framework. The network training program generally consumes substantial time and computational resources [25]. (3) MPS exhibits a strong ability to handle high-dimensional data. Since local patterns play an important role in MPS modeling, it is convenient to expand MPS application to 3D modeling and spatiotemporal simulation. By contrast, the performance of GANs degrades in high-dimensional cases due to the exponential growth of the data space. Aiming at generating realistic structures, it is required to use a specialized network architecture and massive training data.

In this work, we employ Nearest Neighbor Simulation (NNSIM) to motivate MPS stochastic modeling. As an emerging program, the main improvement of NNSIM is to introduce the nearest neighbor searching technique into MPS framework. A range of practical applications indicates that NNSIM exhibits competitive performance in terms of computational efficiency, modeling accuracy and uncertainty quantification. There are three key components within NNSIM. First, prototype selection is used to remove redundant data and compress the training dataset. Based on the Fast Condensed Nearest Neighbor (FCNN) program [26], NNSIM calculates the importance of each training pattern. The instances which have a large distance to the classification boundary are removed to save running time. Second, the teacher–student architecture is designed to control the training dataset. During the modeling procedure, a set of training instances are added to the training dataset. The incorporation of the instance re-sampling strategy has a positive effect on the simulation quality. Third, NNSIM carries out the ball tree program to accelerate the pattern searching program. The training patterns selected by the FCNN and teacher–student step are ranked according to their similarity. A well-organized dataset is helpful for saving redundant computations during the pattern searching step.

### 3.2. Three-Dimensional Modeling Based on Two-Dimensional Training Images

Based on the discussion mentioned above, our program applies MPS to understand the spatial structures of an asphalt mixture sample. The desired patterns are consistently reproduced until there is no uninformed point in the simulation grid. Therefore, the next technical problem is to create a high-dimensional model. In our method, a 2D surface image becomes the prior material to express the spatial structures.

**Figure 9:**
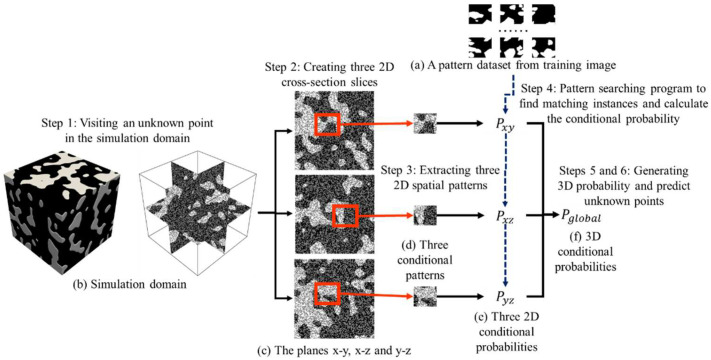
The 3D modeling program on the basis of 2D training image.

One main advantage of MPS is to reconstruct high-dimensional models from limited 2D training images [27]. In this work, we apply the probability aggregation framework. The core idea is to calculate the high-dimensional conditional probability according from multiple 2D cross-sections. Figure 9 displays an illustration of the probability aggregation method. Prior to the 3D modeling step, the pattern dataset is constructed according to the training image. There are six basic steps to realize high-dimensional modeling. (1) The program visits an unknown point in the 3D simulation domain. (2) According to the coordinate of an unknown point, three 2D planes are extracted across multiple cross-sections. In this case, planes x-y, x-z and y-z are independently sampled. (3) For each 2D plane, the program creates a conditioning pattern according to a user-defined template. We adopt a square template in order to simplify the conceptual example. The neighboring known points are used to constitute a conditioning pattern. Therefore, the computer individually creates three 2D conditioning patterns. (4) The pattern searching program is motivated to find similar instances which are presented in the training image. On the basis of the searching result, the center points of suitable training patterns are used to calculate the conditional probability. Since there are three query instances, three conditional probabilities are separately computed according to the 2D conditioning patterns and 2D training image. (5) The probability aggregation method focuses on combining three conditional probabilities. In this work, the Bordley formula is employed. Originating from Bayesian probability theory and the log-linear pooling framework, the Bordley formula addresses the challenges of reconciling conflicts and complementary probabilistic evidence. To preserve the statistical consistency, the odds ratio of each conditional probability is calculated. The Bordley formula performs a sequence of multiplications on odds ratio. Aiming at ensuring reproducibility, Algorithm 1 provides the pseudo-code to implement the Bordley formula. Moreover, the parameter specification plays an important role in the modeling quality. According to the experiment in reference [28], we specify the value of the weight as 0.8. The technical details of the Bordley formula are discussed in reference [28]. (6) Based on the fused probabilities, the program launches a random sampling procedure and predicts the value of unknown points. The preceding steps from (1) to (6) are constantly conducted until each point in the simulation grid has its value.
**Algorithm 1** Probability Aggregation with the Bordley FormulaInput: Training image TI, three 2D conditional probabilities $P_{xy}$, $P_{xz}$ and $P_{yz}$, weight wOutput: 3D conditional probability $P_{global}$
1.  Calculate the prior probability $P_{prior}$ of each facies according to training image TI
2.  Create an empty list Odd = { }
3.  Calculate ${odd\;}_{0}\;=\;P_{prior}/\left(1-P_{prior}\right)$
4.  **for** each probability $P_{i}$ in the list {$P_{prior}$, $P_{xy}$, $P_{xz}$, $P_{yz}$}:
5.   Compute the ${odd\;}_{i}\;=\;P_{i}/\left(1-P_{i}\right)$
6.   Store ${odd\;}_{i}$ into the list Odd
7.  **end**
8.  Calculate the ${odd\;}_{global}$ = ${\left({odd}_{0}\right)}^{1-3w}$
9. **for** each odd term in the list Odd:
10.   ${odd\;}_{global}\;=\;{odd\;}_{global}\times {\left({odd}_{i}\right)}^{w}$
11.   **end**
12.   Calculate the global probability $P_{global}\;=\;{odd}_{global}/\left(1+P_{global}\right)$

**Figure 10:**
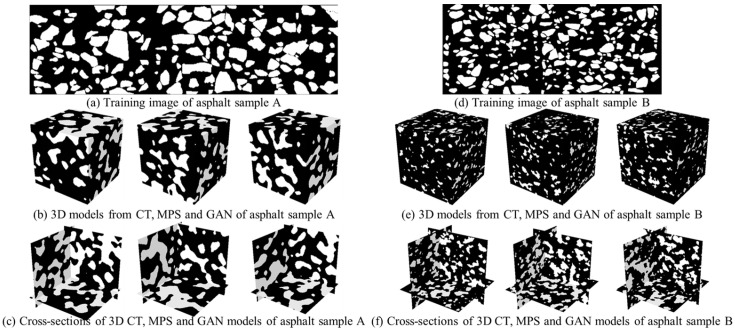
Three-dimensional modeling results from a two-dimensional training image.

Based on the SAM segmentation and MPS reconstruction, we create 20 three-dimensional models from a two-dimensional training image. The size of the 3D model is specified as 128 × 128 × 128. The modeling result is shown in Figure 10. The white area presents the aggregate grain, while the black area expresses the asphalt binder. Moreover, we utilize a CT device to scan the asphalt mixture sample. As a non-destructive method, the CT image has the ability to explicitly express the internal structures. Based on an image thresholding program, the aggregate grains are identified by the distribution of pixel intensities. The 3D CT model shown in Figure 10d becomes the benchmark to examine our imaging, segmentation and modeling method. In addition, we use a Generative Adversarial Network (GAN) as the benchmark method. Based on ref. [29], a generator module associated with six transposed convolutional layers is implemented to create asphalt models. By comparison, the discriminator network is composed of six convolutional layers and max-pooling layers. Due to the absence of a 3D training image, the generator consistently creates 2D images which have matching structures with the 2D training image. To enrich the training dataset, we use MPS to produce 800 2D asphalt images. Then, the GAN network attempts to create a high-dimensional model by using a stack of 2D realizations. To yield realistic results, we specify the training epoch as 200. The GAN result is shown in Figure 10e. Based on visual inspection, there is no substantial difference between the CT model and MPS realizations. In this study, we use a desktop computer with the Windows 10 operating system. The computer used in this study was manufactured by Lenovo Group Limited, Beijing, China, The CPU and GPU configurations are Intel I9-11900 with 2.5 GHz and NVIDIA RTX 3060 with 12G memory. In this asphalt reconstruction case, the NNSIM program costs 0.97 CPU hours to create an individual 3D model. Therefore, the total reconstruction time is 19.4 h. Within the GAN framework, our program consumes 1.87 h to expand the training set. Based on GPU acceleration, the training programs of GAN take 45.4 h to obtain a converged neural network. Then, 0.03 h is used to generate 20 models.

The proposed approach offers four key advancements. First, MPS is a powerful tool to extract and recreate complicated patterns. The pattern reproduction ability is important because the asphalt mixture contains heterogeneous structures. According to the relationship between multiple neighboring points, MPS encourages 3D models to retain morphological dependency in real-world samples. Second, MPS generates diverse 3D realizations for uncertainty quantification. The stochastic characteristics of MPS enable the computer to create a group of 3D realizations. The resulting models not only have matching properties with the input training images but also exhibit large distances to other realizations. For asphalt mixtures, this diversity reflects the variability in aggregate packing, binder distribution and void formation. Based on a diverse model set, it is feasible to support probabilistic prediction of material performance. Third, the pattern searching procedure in NNSIM plays an important role in computational efficiency and running speed. Compared with the deep learning technique, there is no network training or fine-tuning step. Considerable time and resources are saved during the model generation step. Fourth, the integration of the probability aggregation framework reduces the reliance on 3D training images. In practice, it is expensive to yield a 3D model to investigate the intrinsic structure of a porous material. In this work, we synthesize the high-dimensional patterns from a 2D image.

## 4. Numerical Simulation of Geometrical Characteristics and Physical Property

In this section, we focus on evaluating the reconstruction quality of 3D asphalt mixture models. Three metrics are separately implemented. At first, our program applies the statistical characteristics to understand the spatial correlation and dependency. The two-point correlation function is used to compare the training image and generated realizations. Next, we concentrate on the pattern reproduction and uncertainty quantification. Analysis of distance (ANODI) is activated to check the similarity and diversity of the 3D models. Finally, the grain size distribution becomes an important indicator for the modeling accuracy.

It is worth noting that the existing GAN and proposed MPS programs lie in the stochastic modeling framework. In this case, the main objective of GAN and MPS is to create a group of 3D statistically equivalent models which are compatible with the training image. On the one hand, the generated realizations have similar morphological characteristics and physical properties. The spatial structures are continuously extracted from the training image and reproduced into the simulation domain. On the other hand, 3D models exhibit inherent variability in the occurring location, orientation and shape of individual aggregate instance. The diversity and uncertainty between 3D asphalt models become important aspects of the reconstruction accuracy. In other words, the stochastic modeling technique is not designed to produce a perfect pixel-wise alignment with the CT model. Therefore, our validation strategy focuses on capturing the essential characteristics from training images, the CT model and the reconstruction results.

### 4.1. Modeling Quality Evaluation Based on Statistical Characteristics

The key to modeling accuracy lies in replicating the geometric and topological characteristics of the training images. Therefore, the consistency between 2D training images and 3D asphalt mixture realizations attracts our attention. In this work, we employ two-point statistics to check the statistical characteristics of the numerical models. As a widely used method, the statistical functions provide a robust framework for evaluating the reconstructed models, preserving spatial continuity, connectivity and structural patterns.

As its name implies, the two-point correlation function is a fundamental tool to assess the spatial arrangement within the porous models [30]. Given two points with a specified distance and direction, this method quantifies the probability that two points are allocated into the same phase. In this case, we focus on the spatial dependency of aggregate grains. Therefore, the white area in the asphalt mixture becomes the target variable. For the scientific evaluation, we compute the two-point correlation function for both the 2D training image and 3D realizations. Along the fixed direction, a collection of points is sampled at varying distances. To ensure a comprehensive comparison, we extract 2D slices from 3D realizations across multiple cross-sections. Therefore, the two-point statistics are directly compared with the 2D training image. A close agreement between two correlation functions indicates that MPS is capable of reproducing the short- and long-range dependency in the training image. Since the asphalt mixture sample is created by being completely stirred, the two-point statistics function is effective for evaluating the homogeneity of the aggregate grain.

**Figure 11:**
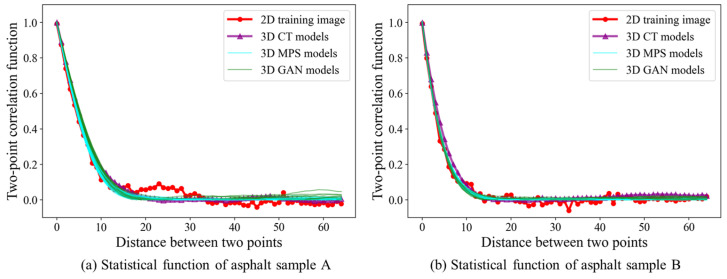
Quality check with two-point correlation function.

The calculation of the two-point correlation function is shown in Figure 11. The red curve indicates the spatial dependency of the 2D training image. By comparison, the spatial correlation of the CT model is highlighted by purple. The two-point correlation functions of the 3D MPS models are represented by multiple blue lines. It is clear that the correlation functions exhibit similar tendencies. Thus, our stochastic modeling program is able to reproduce the spatial structure from 2D images in the 3D domain.

### 4.2. Modeling Uncertainty Quantification Based on Analysis of Distance

In the context of stochastic modeling, there are two main factors to evaluate the reconstruction accuracy. On the one hand, the computer program repeatedly extracts spatial patterns from the training image and reproduces suitable structures in the simulation domain. Therefore, the generated realization should have a strong similarity to the training image. On the other hand, diversity and variability are important to reflect the inherent uncertainty during the material production step. There is an intensive difference between two 3D realizations. Thus, the previous conflicting metrics brings a big challenge to the modeling and reconstruction program.

**Figure 12:**
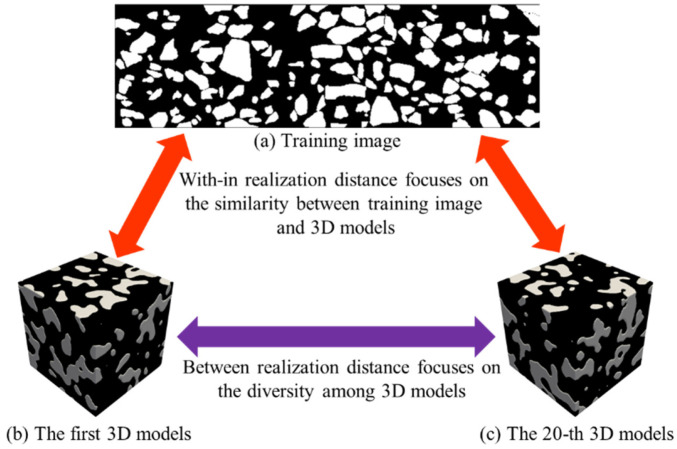
The core idea of ANODI in the context of stochastic modeling.

In this work, we apply analysis of distance (ANODI) to assess the MPS models. The core idea of ANODI is shown in Figure 12. Rather than two-point statistics, ANODI focuses on the probability distribution of spatial patterns. A pattern histogram is created to express the key structures. Then, the Jensen–Shannon divergence is employed to calculate the similarity between two distributions. A small distance indicates there is a slight difference between the two realizations. Finally, ANODI conducts a manifold learning approach to visualize the calculation result. Based on the distance matrix, the high-dimensional models are projected into a 2D feature space. In the feature space, one point represents an individual model. Two close points indicate that there is a high agreement between two material models. A wide point dispersal reveals that the model group is able to express adequate variability.

In accordance with the previous discussion, the pattern histogram is at the heart of ANODI calculation. There are six basic steps to characterize the 2D and 3D images. (1) Given a specified training image, a template is applied to extract spatial patterns from the training image. In general, a template of size 7 × 7 is widely used. Based on the sliding window strategy, the program captures a range of pattern instances. (2) ANODI launches a clustering procedure to organize training patterns. Similar patterns are allocated into the same group. By comparison, the instances in different groups imply that there is a big mismatch. (3) For each group, the evaluation program calculates the number of member instances. A frequency distribution is created to express the occurrence of each pattern group. Moreover, ANODI records the centroid of each group as the representative examples. (4) On the basis of a generated realization, the template and sliding window strategy are used to extract spatial patterns. (5) The spatial pattern is independently allocated according to its distance to the representative instances. The computer conducts a clustering method to organize the patterns in the generated realizations. (6) The program counts the number of member instances. A pattern histogram is produced as the feature vector of a generated realization.

The evaluation step in ANODI involves two key distance calculations. First, the within-realization distance measures the agreement between each individual realization and the training images. A low within-realization distance indicates that the reconstructed realization is successful at reproducing spatial patterns from the training image to the simulation domain. Second, the between-realization distance attempts to quantify the variability among the ensemble of realizations. This distance metric is calculated as the average Jensen–Shannon divergence between every pair of generated realizations. A high value of the between-realization distance is preferred because there is sufficient diversity in the generation results.

**Figure 13:**
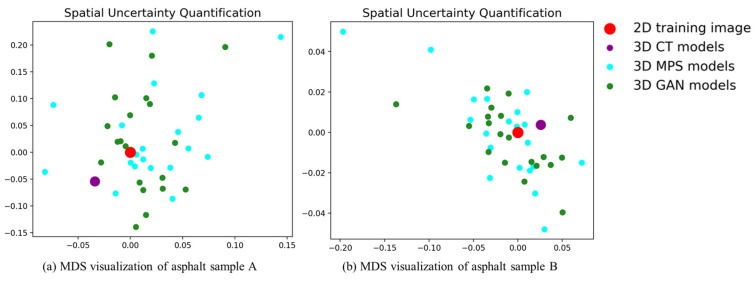
MDS visualization results based on ANODI.

Based on the MPS realizations, we conduct ANODI to quantify the pattern reproduction and spatial uncertainty. For the first asphalt sample, the within-realization distance is 0.14. The value of the between-realization distance is 0.30. By comparison, the two distances of the GAN models are 0.13 and 0.27, respectively. On the other hand, the distances of the MPS models for the second asphalt sample reach 0.06 and 0.11. In contrast, the GAN program obtains 0.05 and 0.09 when ANODI calculates the within-realization and between-realization distances. A notable phenomenon is that the value of the Jensen–Shannon divergence is bounded by one for two probability distributions. Therefore, the calculation result indicates that MPS has comparable reconstruction quality to GAN. Furthermore, the visualization of ANODI is shown in Figure 13. Multi-dimensional scaling (MDS) is performed to realize the manifold learning step. In this 2D plot, one point expresses a 2D image or 3D model. Red, purple and blue are used to indicate the distribution of the 2D asphalt mixture image, 3D CT model and MPS realizations, respectively. Obviously, the red and purple points are surrounded by the blue points. This cloud dispersal implies that the spatial structures of the asphalt mixture sample are reconstructed by the MPS programs. Therefore, our method exhibits competitive performance in creating an ensemble of high-quality 3D models.

### 4.3. Physical Property Estimation Based on Aggregation Grain Distribution

In the previous sections, we applied statistical characteristics and ANODI to assess the pattern reproduction ability and spatial uncertainty. It is meaningful for ensuring that the MPS reconstruction program preserves the key structural attributes and physical properties. In this work, the distribution of aggregate grain attracts our attention. The grain distribution is a decisive factor to porosity, permeability and mechanical behavior in porous materials. There are three key steps to realize the extensive evaluation. At first, each aggregate particle is individually labeled in the 3D realizations. Next, the size of each aggregate grain is calculated as the target property. Finally, the grain size distribution is compared with a benchmark value to quantify the modeling accuracy.

To compute the bounding sphere of each aggregate extracted from SAM, the Extremal Points Optimal Sphere (EPOS) [31] algorithm is adopted. EPOS offers a controllable trade-off between computational speed and accuracy in solving the bounding sphere problem. The EPOS algorithm is composed of four steps. First, a set of direction vectors is selected. Second, all points in an individual aggregate grain are projected along each direction vector. EPOS identifies the extremal points with the minimum and maximum projection values in each direction. This projection operation results in a candidate set of bounding points. Third, a precise minimum bounding sphere is computed from these extremal points using an exact solver. Fourth, the remaining points are examined to refine the calculation result. If any points lie outside the current sphere, the algorithm iteratively expands the sphere with the aim of ensuring all points are enclosed. Compared to other bounding sphere extraction methods, the EPOS approach significantly reduces computational time while maintaining a high level of accuracy. In this study, the gradation of aggregates is evaluated using a standard set of sieve sizes: 0.075 mm, 0.15 mm, 0.3 mm, 0.6 mm, 1.18 mm, 2.36 mm, 4.75 mm, 9.5 mm, 13.2 mm and 16 mm.

CT scanning was performed using a system equipped with a CCD sensor of 2048 × 2048 pixels, resulting in an image voxel size of approximately 54 μm. Consequently, aggregates smaller than 0.075 mm could not be directly observed in the CT images. Instead, the content of aggregates below 0.075 mm was determined based on the original aggregate gradation employed during specimen preparation.

**Figure 14:**
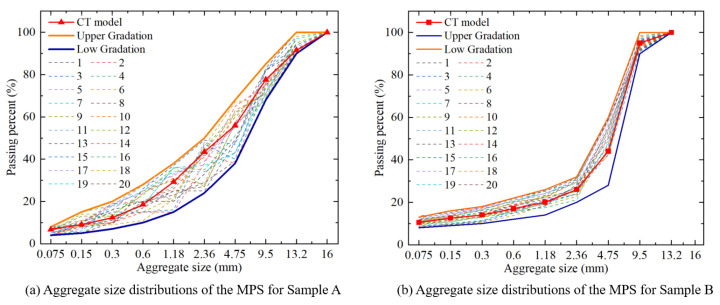
Aggregate size distributions of asphalt mixture sample and MPS realizations.

Figure 14 presents the aggregate size distributions of the CT model and the MPS realizations based on mass-based gradation. Figure 14a shows the size distributions for 20 MPS realizations of AC-13, while Figure 14b presents the results for 20 realizations of AC-10. All aggregate size distributions of these realizations are within the specified upper and lower limits. The results confirm the robustness and effectiveness of MPS. Each realization satisfies the specified requirements, demonstrating not only the accuracy of the 3D reconstruction method but also its ability to generate multiple reliable virtual specimens. The MPS method effectively captures the variability in aggregate size distributions while ensuring compliance with design specifications, reinforcing its practical applicability in simulating real-world asphalt pavement materials.

To further support the statistical similarity between the generated samples and the CT-based reference, we perform a Kolmogorov–Smirnov (KS) test on all MPS realizations. As shown in Table 2, the p-values of the KS tests are consistently above 0.05, indicating no statistically significant difference between the aggregate size distributions of the generated samples and the reference model.

**Figure 15:**
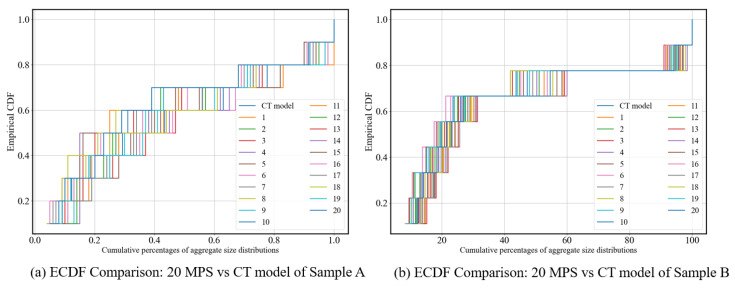
**E**CDF comparison: 20 MPS realizations vs. CT model.

Figure 15 presents the Empirical Cumulative Distribution Function (ECDF) comparison between the CT model and the 20 MPS realizations samples under AC-13 (Figure 15a) and AC-10 (Figure 15b) conditions. Based on the ECDF visualization and KS test analysis, it can be observed that the MPS realization samples generally exhibit a favorable agreement with the CT model gradation curves. For AC-10, the MPS realizations show consistent trends across the entire aggregate size spectrum. In contrast, for AC-13, although greater variability is observed in the coarse aggregate range, the samples demonstrate better convergence in the fine aggregate region. Overall, MPS shows strong capability in reconstructing gradation profiles, particularly excelling in representing the distribution.

## 5. Conclusions

In this paper, we explore a low-cost and data-efficient way to create 3D asphalt mixture models. The core idea is to integrate the strengths of the foundational segmentation program and stochastic modeling. There are three fundamental modules within our method. First, the 2D image is collected to express the spatial structures of an asphalt mixture sample. Based on a smartphone-based system and image quilting program, our program gathers a 2D image with fine-grained details and complete coverage. Without the network training and image labeling procedures, the Segment Anything Model (SAM) is launched to distinguish the aggregate grain and asphalt binder. Robust aggregate segmentation is achieved by the strong generalization ability of the foundational SAM network. Second, we use Multiple-Point Statistics (MPS) to build 3D asphalt mixture models from 2D training images. To save running time, Nearest Neighbor Simulation (NNSIM) is performed to accelerate the pattern searching program. A probability aggregation framework is introduced to generate high-dimensional conditional probability according to several 2D cross-sections. Third, the model quality evaluation step is conducted to examine the generated realizations. Statistical characteristics, analysis of distance (ANODI) and particle size distribution are independently employed to check the 3D models. The calculation results indicate that our program exhibits competitive performance in terms of pattern reproduction and uncertainty quantification. The accurate 3D models become a promising tool to understand the complex relationship between spatial structures and physical properties.

It should be noted that the main objective of our method is to improve data accessibility from the smartphone-based system and facilitate 3D stochastic modeling via MPS. Therefore, the proposed 3D reconstruction framework utilizes an individual 2D surface image to express the prior knowledge. An underlying assumption is that the asphalt mixture sample is isotropic and stationary. For anisotropic material and geometries with high aspect ratios, future work will explore the integration of cross-sectional images and directional statistical information. It is meaningful to introduce anisotropic characteristics into the MPS reconstruction framework. In addition, we use the SAM program to deal with asphalt images. However, SAM struggles with occluded grains in 2D images. It is meaningful to enhance the segmentation process by incorporating domain-specific knowledge. Moreover, the illumination settings and camera configuration play an important role in image quality. The method is limited to reconstructing the aggregate phase, as with smartphone imaging, it is difficult to obtain explicit information about voids. Thus, future research will focus on the robustness of the imaging system. For example, it is meaningful to introduce adaptive illumination normalization and shadow removal programs. Deep-learning-based super-resolution methods are useful to address the limitations of low-resolution images.

## Figures and Tables

**Table 1:** Quantitative evaluation of segmentation results.

Sample	Segmentation Method	Pixel Accuracy	Intersection of Union	Dice Coefficient	Hausdorff Distance
Sample A	SAM	0.9456	0.8830	0.9379	7.0711
	Ostu thresholding	0.8003	0.6402	0.7807	8.0000
	Adaptive thresholding	0.7650	0.6264	0.7703	9.4868
Sample B	SAM	0.9442	0.8525	0.9204	20.6155
	Ostu thresholding	0.7749	0.6016	0.7513	35.3836
	Adaptive thresholding	0.6870	0.4946	0.6619	34.6699

**Table 2:** KS test statistics and p-values of MPS realizations for samples vs. CT model.

Index	MPS for Sample A		MPS for Sample B	
	Statistic	*p*-Value	Statistic	*p*-Value
1	0.3133	0.2769	0.2129	0.3807
2	0.3026	0.3146	0.1479	0.6589
3	0.3226	0.2472	0.1787	0.5532
4	0.3188	0.2589	0.1670	0.6015
5	0.3479	0.1777	0.1258	0.6912
6	0.3169	0.2650	0.1853	0.5222
7	0.3185	0.2599	0.1357	0.6808
8	0.321	0.2521	0.1811	0.5423
9	0.2965	0.3377	0.1506	0.6526
10	0.3552	0.1605	0.1677	0.5989
11	0.3245	0.2414	0.1400	0.6743
12	0.3288	0.2284	0.1303	0.6872
13	0.3148	0.2720	0.1978	0.4601
14	0.3282	0.2303	0.2334	0.2705
15	0.2825	0.3951	0.2380	0.2462
16	0.3492	0.1746	0.1414	0.6719
17	0.3159	0.2684	0.1164	0.6965
18	0.3215	0.2506	0.2018	0.4396
19	0.3228	0.2463	0.1460	0.6631
20	0.3207	0.2530	0.1343	0.6827

## Data Availability

The original contributions presented in this study are included in the article. Further inquiries can be directed to the corresponding author.
